# Eight months follow-up study on pulmonary function, lung radiographic, and related physiological characteristics in COVID-19 survivors

**DOI:** 10.1038/s41598-021-93191-y

**Published:** 2021-07-05

**Authors:** Shengding Zhang, Wenxue Bai, Junqing Yue, Lu Qin, Cong Zhang, Shuyun Xu, Xiansheng Liu, Wang Ni, Min Xie

**Affiliations:** 1 Department of Respiratory and Critical Care MedicineTongji Hospital, Tongji Medical College, Huazhong University of Science and Technology, Wuhan, 430030 China; 2 Key Laboratory of Respiratory Diseases, National Ministry of Health of the People’s Republic of China and National Clinical Research Center for Respiratory Disease, Wuhan, China

**Keywords:** Diseases, Infectious diseases, Respiratory tract diseases

## Abstract

To describe the long-term health outcomes of patients with COVID-19 and investigate the potential risk factors. Clinical data during hospitalization and at a mean (SD) day of 249 (15) days after discharge from 40 survivors with confirmed COVID-19 (including 25 severe cases) were collected and analyzed retrospectively. At follow-up, severe cases had higher incidences of persistent symptoms, DLCO impairment, and higher abnormal CT score as compared with mild cases. CT score at follow-up was positively correlated with age, LDH level, cumulative days of oxygen treatment, total dosage of glucocorticoids used, and CT peak score during hospitalization. DLCO% at follow-up was negatively correlated with cumulative days of oxygen treatment during hospitalization. DLCO/VA% at follow-up was positively correlated with BMI, and TNF-α level. Among the three groups categorized as survivors with normal DLCO, abnormal DLCO but normal DLCO/VA, and abnormal DLCO and DLCO/VA, survivors with abnormal DLCO and DLCO/VA had the lowest serum IL-2R, IL-8, and TNF-α level, while the survivors with abnormal DLCO but normal DLCO/VA had the highest levels of inflammatory cytokines during hospitalization. Altogether, COVID-19 had a greater long-term impact on the lung physiology of severe cases. The long-term radiological abnormality maybe relate to old age and the severity of COVID-19. Either absent or excess of inflammation during COVID-19 course would lead to the impairment of pulmonary diffusion function.

## Introduction

Coronavirus disease 2019 (COVID-19) is a recently emerged infectious disease caused by severe acute respiratory syndrome coronavirus 2 (SARS-CoV-2)^1^. As of December 31, 2020, accumulative 81,475,053 confirmed cases and 1,798,050 confirmed deaths were reported globally. During the outbreak period, researchers mainly focus on the epidemiological characteristics, infection and pathophysiological mechanisms, as well as treatment methods of this disease. Previous studies have shown that COVID-19 involves multiple organs, and the lung is one of the most important organs involved.

Among patients with COVID-19, about 14% cases were severe and 5% cases were critical^2^, and the overall case-fatality rate (CFR) was 5.0% (4788 total deaths/96,673 total confirmed cases) in China, based on data up to December 31, 2020, from Chinese Center for Disease Control and Prevention. Although the overall CFR of COVID-19 is lower than that of severe acute respiratory syndrome (SARS) (9.6%) and Middle East respiratory syndrome (MERS) (34.4%)^2^, radiology and lung function abnormalities can be found in a considerable proportion of COVID-19 survivors at time of hospital discharge, in early convalescence phase, and even at three and six months after discharge^3–7^.Severe patients had a higher incidence of diffusion capacity of the lung for carbon monoxide (DLCO) impairment and encountered more total lung capacity (TLC) decrease and six-minute walk distance (6MWD) decline compared with non-severe patients at 30 days after discharged^4^. While there is lack of clinical evidence for the long-term follow up of pulmonary function and physiological disorder in severe COVID-19 patients.

Thus, we retrospectively collected and analyzed the clinical data during hospitalization and at eight months after discharge to investigate the long-term impact of severe COVID-19 on pulmonary function, chest high-resolution computed tomography (HRCT) pictures, and related physiological characteristics and try to find out the potential risk factors.

## Results

### Subjects’ characteristics

We recruited 21 women and 19 men with a media (IQR) age of 57 (40–68) years and a mean ± SD body mass index (BMI) of 25.47 ± 4.22 kg/m^2^ including 25 severe cases. Common comorbidities included hypertension (18 cases, 45.0%), diabetes mellitus (six cases, 15.0%), coronary heart disease (four cases, 10.0%), chronic obstructive pulmonary disease (COPD) (two cases, 5.0%), and asthma (one case, 2.5%). Eight (20.0%) patients were smokers, but only one was current smoker on admission. During the period having COVID-19, 32 (80.0%) patients received oxygen treatment and two of them required additional noninvasive positive pressure ventilation (NIPPV) treatment and ICU admission; 24 (60.0%) patients received glucocorticoids and five of them required high-dose glucocorticoids.

**Figure 1:**
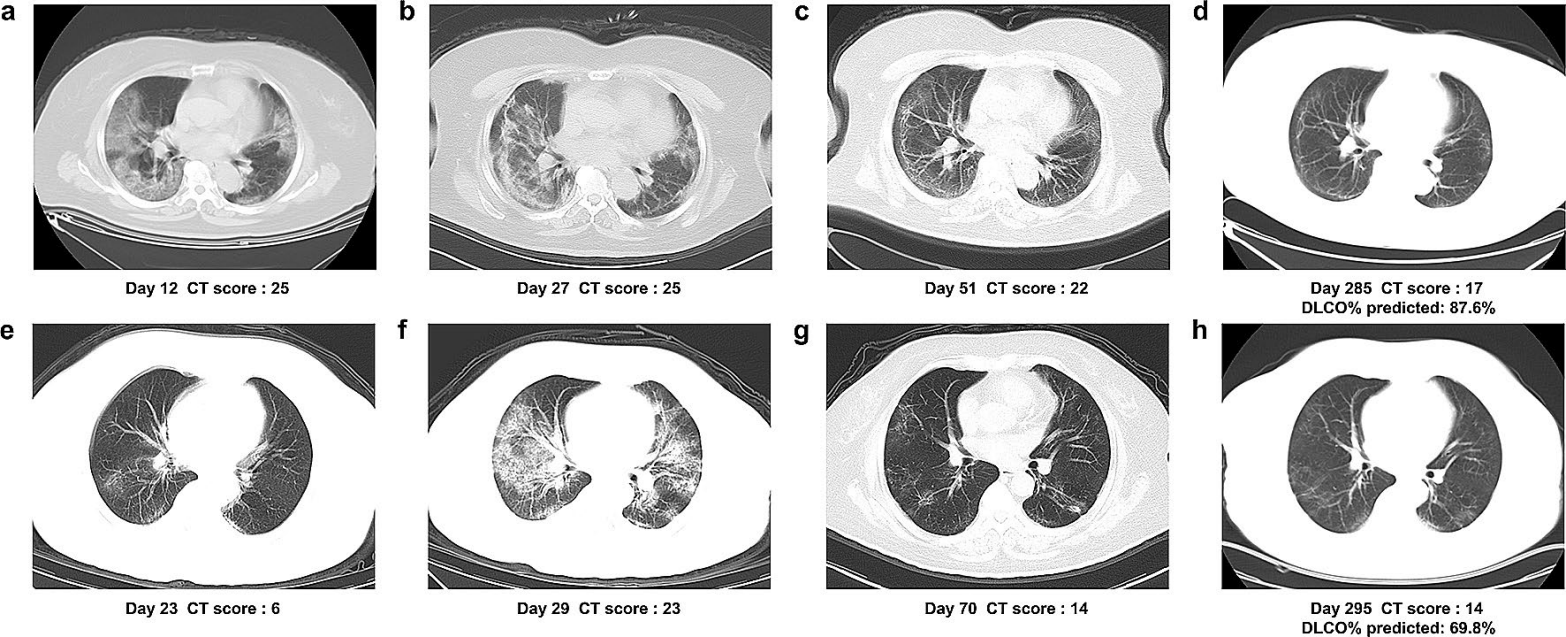
Serial HRCTs of two severe COVID-19 with different pulmonary diffusion function status. (**a**–**d**) Serial HRCTs of a 65-year-old COVID-19 female patient with normal pulmonary diffusion function at eight months post discharge: (**e**–**h**) Serial HRCTs of a 71-year-old COVID-19 female patient with abnormal pulmonary diffusion function at eight months post discharge.

**Figure 2:**
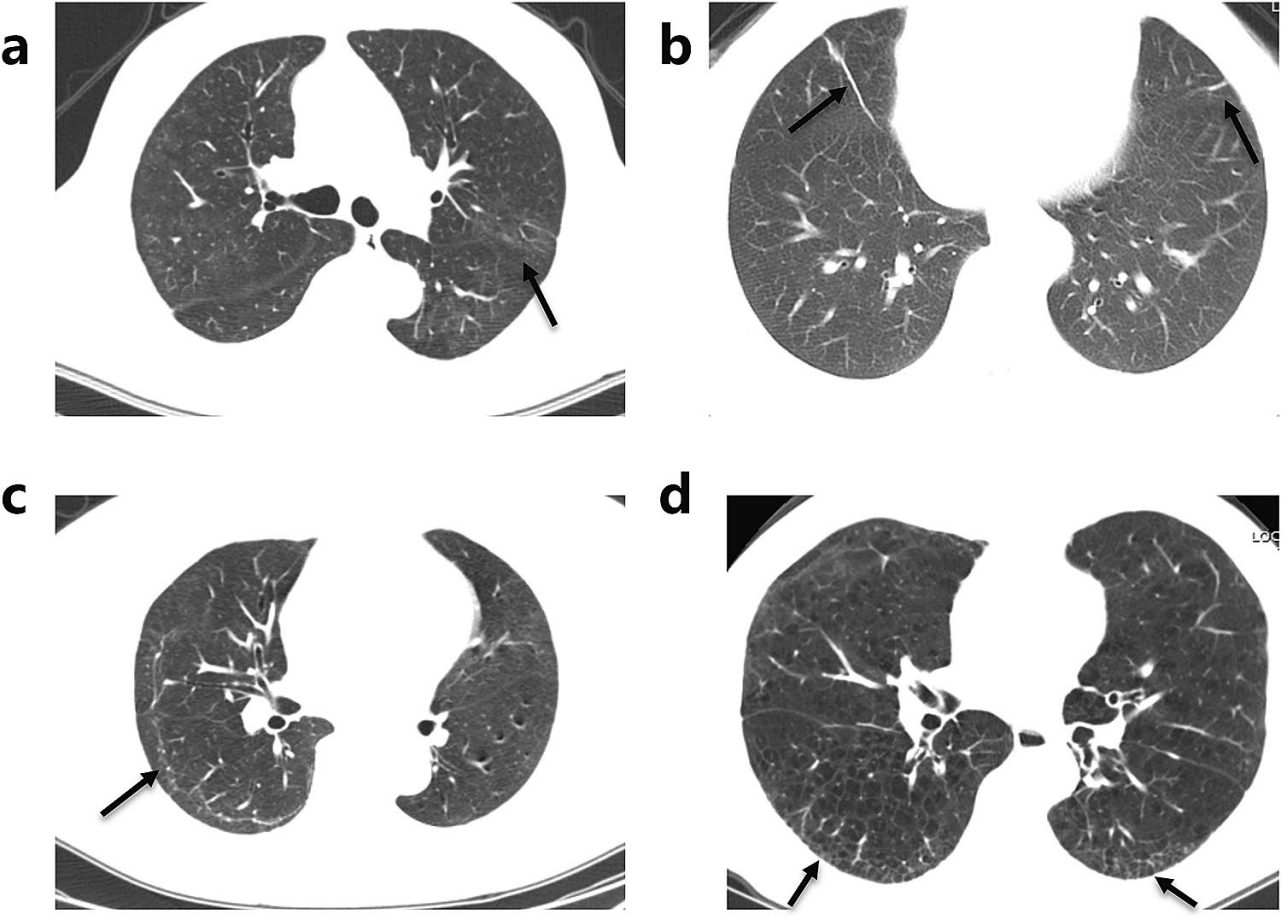
Abnormal CT patterns of severe COVID-19 patient at follow-up of eight months post discharge. The abnormal CT patterns are indicated by arrows in (**a**) ground glass opacities, (**b**) irregular lines, (**c**) subpleural line, and (**d**) reticular pattern which could still be seen on some of COVID-19 patients in convalescence of eight months post discharge.

The follow-up was obtained on an average ± SD of 249 ± 15 days after discharge. At eight months 22 (55.0%) patients still had persistent physical and (or) psychological symptoms, and nine (22.5%) patients still suffered from different degrees of limitations in daily life. Although all patients had normal FVC, one (2.5%), 13 (32.5%), and nine (22.5%) patients had TLC, DLCO, and DLCO/VA below 80% of predicted values, respectively. Eight (20.0%) patients had FEV_1_/FVC below 70% of predicted values, and two of them had history of COPD with significant history of cigarette smoking, the other six had no history of COPD, asthma, or cigarette smoking. Twenty-two (55.0%) patients had at least two of the three indexes of MEF50, MEF25, and MMEF75/25 below 65% of predicted values, which indicates small airway dysfunction. The chest HRCT of 28 (70.0%) patients were normal or basically normal (CT score < 5), whereas the remaining 12 patients had an abnormal CT at eight months after discharge. However, CT abnormalities and pulmonary function impairments were not completely consistent on all of patients. Follow-up CT images of two severe COVID-19 patients with abnormal CT but different pulmonary diffusion function status at eight months after discharge were presented in Fig. 1. Most common abnormal CT patterns were ground glass opacity (GGO) (21 cases, 52.5%), irregular lines (19 cases, 47.5%), subpleural line (two cases, 5.0%), and reticular pattern (two cases, 5.0%) (Fig. 2).

### Subgroup analyses

#### Patients were divided into two groups according to severity of COVID-19

In compared with mild cases, severe cases had higher CT peak score (*p* < 0.001), higher rate of receiving oxygen treatment (*p* = 0.007), longer cumulative days of oxygen treatment (*p* < 0.001), higher maximum inhaled oxygen concentration (*p* = 0.004), and longer cumulative days of receiving glucocorticoids during hospitalization (*p* = 0.020), a trend of higher rate of receiving glucocorticoids (*p* = 0.094), and a trend of higher total dosage of glucocorticoids used during hospitalization (*p* = 0.054). More details were summarized in Table 1.

**Table 1:** Demography and treatment of participants by the severity of COVID-19.

General data	Mild cases (n = 15)	Severe cases (n = 25)	*p* value
Age (years)	57 (38–72)	57 (45–66)	0.771
Gender (female/male)	6/9	15/10	0.328
BMI, kg/m^2^	24.44 ± 2.81	26.09 ± 4.82	0.238
CT peak score £	5 (4–12)	17 (10–23)	** < 0.001**
**Smoking status, n (%)**			
Never	12 (80%)	20 (80%)	> 0.999
Former	3 (20%)	4 (16%)	
Current	0 (0%)	1 (4%)	
**Comorbidities, n (%)**			
COPD	0 (0%)	2 (8.0%)	0.519
Asthma	0 (0%)	1 (4.0%)	> 0.999
Diabetes mellitus	0 (0%)	6 (24.0%)	0.067
Hypertension	7 (46.7%)	11 (44.0%)	> 0.999
Coronary heart disease	0 (0%)	4 (16.0%)	0.278
**Treatment received during hospitalization**			
Oxygen treatment, n (%)	9 (60.0%)	24 (96.0%)	**0.007**
Cumulative days of oxygen treatment, d (13 vs 25)	2 (0–17)	26 (15–35)	** < 0.001**
Maximum inhaled oxygen concentration, % (13 vs 22)	29 (21–41)	41 (33–54)	**0.004**
NIPPV, n (%)	0 (0%)	2 (8.0%)	0.519
Glucocorticoids, n (%)	6 (40.0%)	18 (72.0%)	0.094
Cumulative days of receiving glucocorticoids, d (15 vs 24)	0 (0–7)	10 (0–12)	**0.020**
Total dosage of glucocorticoids, mg‡ (15 vs 24)	0 (0–400)	350 (0–538)	0.054
Antiviral treatment, n (%)	13 (86.7%)	23 (92.0%)	0.623
Antibiotic treatment, n (%)	11 (73.3%)	22 (88.0%)	0.392
Immunoglobulin therapy, n (%)	4 (26.7%)	10 (40.0%)	0.502
ICU admission, n (%)	0 (0%)	2 (8.0%)	0.519

Data were expressed as mean ± SD, median (interquartile range), or No. (%). Comparisons were determined by Student’s t-test, Mann–Whitney U test or Fisher exact tests as appropriate. The actual number of cases was marked behind each index, when there was missing data.

*BMI* body mass index at eight months after discharge, *COPD* chronic obstructive pulmonary disease, *NIPPV*, noninvasive positive presure ventilation, *ICU* intensive care unit.

^£^The CT peak score during hospitalization of one patient with severe COVID-19 was missing.

^‡^Total dosage of glucocorticoid means accumulative dosage of glucocorticoid converted to prednisone for COVID-19 patients.

At eight months after discharge, more patients in severe cases group had persistent physical and (or) psychological symptoms (*p* = 0.009). Severe cases had higher score in PF (*p* = 0.043), higher rate of abnormal DLCO (*p* = 0.013), higher rate of small airway dysfunction (*p* = 0.050), lower FEV_1_% predicted (*p* = 0.008), lower FVC% predicted (*p* = 0.042), lower MEF50% predicted (*p* = 0.023), lower MVV% predicted (*p* = 0.032), higher Z at 5 Hz% predicted (*p* = 0.020), which indicated higher total respiratory impedance, higher Rperipheral (*p* = 0.015), which indicated higher resistance in the peripheral airways, lower DLCO% predicted (*p* = 0.023), and higher CT score (*p* = 0.009) in compared with mild cases. More details were summarized in Table 2.

**Table 2:** Pulmonary function, chest CT, and related physiological characteristics of COVID-19 patients at eighth months of follow-up.

Parameters	Mild cases (n = 15)	Severe cases (n = 25)	*p* value
**PCFS scale grade, n (%)**			
PCFS scale grade ≥ 1	4 (26.7%)	18 (72%)	**0.009**
PCFS scale grade ≥ 2	3 (20.0%)	6 (24%)	0.311
**SF-36**			
PF	100 (85–100)	90 (80–95)	**0.043**
RP	100 (100–100)	100 (38–100)	0.594
BP	100 (84–100)	74 (73–100)	0.109
GH	70 (55–97)	72 (57–92)	0.928
VT	90 (65–95)	80 (70–90)	0.582
SF	75 (63–100)	75 (63–100)	0.972
RE	100 (100–100)	100 (50–100)	0.607
MH	84 (76–88)	80 (60–92)	0.482
HT	50 (25–50)	50 (25–50)	0.744
PCS	92 (61–99)	82 (68–92)	0.193
MCS	88 (79–94)	83 (61–95)	0.725
**Pulmonary function**			
FVC < 80% of predicted values, n (%)	0 (0%)	0 (0%)	> 0.999
FEV_1_/FVC < 70% of predicted values, n (%)	2 (13.3%)	6 (24.0%)	0.686
MVV < 80% of predicted values, n (%)	0 (0%)	1 (4.0%)	> 0.999
TLC < 80% of predicted values, n (%)	0 (0%)	1 (4.0%)	> 0.999
DLCO < 80% of predicted values, n (%)	1 (6.7%)	12 (48.0%)	**0.013**
DLCO/VA < 80% of predicted values, n (%)	2 (13.3%)	7 (28.0%)	0.440
At least two of the three indexes of MEF50, MEF25, and MMEF75/25 < 65% of predicted values, n (%)	5 (33.3%)	17 (68.0%)	**0.050**
FEV_1_% predicted	112.3 ± 13.6	98.2 ± 16.4	**0.008**
FVC% predicted	121.3 ± 16.0	110.8 ± 14.7	**0.042**
FEV_1_/FVC, %	75.4 ± 5.1	73.7 ± 10.5	0.570
PEF% predicted	114.5 ± 20.0	108.1 ± 16.5	0.282
MEF50% predicted	83.2 ± 19.5	66.2 ± 23.2	**0.023**
MEF25% predicted	53.5 ± 14.3	48.9 ± 28.0	0.556
MMEF75/25% predicted	71.5 ± 16.5	57.3 ± 25.7	0.064
MVV% predicted	119.7 ± 13.8	107.7 ± 10.1	**0.032**
Z at 5 Hz% predicted	88.5 ± 17.6	105.0 ± 22.5	**0.020**
Rperipheral, kPa/(L/s)	0.18 ± 0.11	0.26 ± 0.09	**0.015**
X at 5 Hz corrected by predicted value, kPa/(L/s)	-0.07 ± 0.03	-0.08 ± 0.05	0.490
TLC% predicted	100.9 ± 6.8	95.0 ± 9.4	**0.042**
RV% predicted	93.9 ± 12.7	99.1 ± 22.2	0.410
RV/TLC, %	33.1 ± 8.1	37.3 ± 6.5	0.078
DLCO% predicted	94.1 ± 14.6	82.6 ± 14.8	**0.023**
DLCO/VA% predicted	95.8 ± 13.5	89.9 ± 14.8	0.206
**Chest HRCT abnormalities**			
GGO, n (%)	5 (33.3%)	16 (64.0%)	0.102
Irregular lines, n (%)	7 (46.7%)	12 (48.0%)	> 0.999
Consolidation, n (%)	0 (0%)	0 (0%)	> 0.999
Interlobular septal thickening, n (%)	0 (0%)	0 (0%)	> 0.999
Subpleural line, n (%)	0 (0%)	2 (8.0%)	0.519
Reticular pattern, n (%)	1 (6.7%)	1 (4.0%)	> 0.999
CT score	0 (0–3)	3 (2–10)	**0.009**
Abnormal HRCT (CT score ≥ 5), n (%)	2 (13.3%)	10 (40.0%)	0.152
6MWD, m	558 ± 80	543 ± 88	0.583
Time from discharge to follow-up, d	247 ± 11	251 ± 17	0.402

Data were expressed as mean ± SD, median (interquartile range), or No. (%). Comparisons were determined by Student’s t-test, Mann–Whitney U test or Fisher exact tests as appropriate. The actual number of cases was marked behind each index, when there was missing data.

*BMI* body mass index, *PCFS scale* Post-COVID-19 Functional Status scale, *PF* physical functioning, *RP* role limitation due to physical problems, *BP* bodily pain, *GH* general health, *VT* vitality, *SF* social functioning, *RE* role limitation due to emotional problems, *MH* mental health, *HT* reported health transition, *PCS* physical component summary, *MCS* mental component summary, *FVC* forced vital capacity, *FEV*_*1*_ forced expiratory volume in one second, *MVV* maximum voluntary ventilation, *TLC* total lung capacity, *DLCO* diffusion capacity of the lung for carbon monoxide, *DLCO/VA* ratio of carbon monoxide diffusion capacity to alveolar ventilation, *MEF50* maximal expiratory flow at 50% of FVC, *MEF25* maximal expiratory flow at 25% of FVC, *MMEF75/25* maximal midexpiratory flow between 75 and 25% of FVC, *Z at 5 Hz* impedance at 5 Hz, which indicates the total respiratory impedance, *Rperipheral* resistance in the peripheral airways, *X at 5 Hz* reactance at 5 Hz corrected by predicted value, which inversely indicates the elastic recoil of the peripheral airways, *HRCT* high-resolution computed tomography, *GGO* ground glass opacity, *6MWD* 6-min walk distance.

#### Patients were divided into two groups according to CT score at follow up

As compared with patients with normal or basically normal CT (CT score < 5) at eight months, patients with abnormal CT (CT score ≥ 5) had higher CT peak score (*p* < 0.001), lower lymphocyte count (*p* = 0.003), higher procalcitonin (PCT) level (*p* = 0.038), higher lactate dehydrogenase (LDH) level (*p* = 0.021), higher D-dimer level (*p* = 0.031), lower albumin level (*p* = 0.002), higher aspartate aminotransferase (AST) level (*p* = 0.032), longer cumulative days of oxygen treatment (*p* < 0.001), higher maximum inhaled oxygen concentration (*p* = 0.010), longer cumulative days of receiving glucocorticoids (*p* = 0.007), and higher total dosage of glucocorticoids used during hospitalization (*p* = 0.024).

At eight months after discharge, patients with abnormal CT had higher BMI (*p* = 0.019), lower TLC% predicted (*p* = 0.005), lower RV% predicted (*p* = 0.007), and higher rate of abnormal DLCO (*p* = 0.032). For abnormal CT patterns, patients with abnormal CT were more likely to have residual GGO (*p* = 0.002) and irregular lines (*p* = 0.005). More details were summarized in Table 3 and Supplementary Table S1.

**Table 3:** Characteristics of participants according to CT scanning at follow-up.

Parameters	CT score at eight months after discharge		*p* value
	< 5 (n = 28)	≥ 5 (n = 12)	
Age (years)	51 (38–68)	65 (55–65)	0.256
Gender (female/male)	15/13	6/6	> 0.999
CT peak score £	9 (5–14)	23 (14–24)	** < 0.001**
Severe cases, n (%)	15 (53.6%)	10 (83.3%)	0.152
**Laboratory data during hospitalization**			
Lymphocyte count min (× 10^9^/L) _(27 vs 12)_	1.08 (0.73–1.50)	0.77 (0.46–0.93)	**0.003**
Eosinophil count min (× 10^9^/L) _(26 vs 12)_	0.01 (0–0.03)	0 (0–0.02)	0.309
IL-2R peak, U/ml _(17 vs 10)_	536 (440–755)	792 (498–1109)	0.183
IL-6 peak, pg/ml _(18 vs 10)_	4.20 (2.40–25.79)	8.89 (3.20–39.15)	0.493
IL-8 peak, pg/ml _(17 vs 10)_	15.40 (7.90–43.70)	16.85 (11.80–30.38)	0.776
TNF-α peak, pg/ml _(17 vs 10)_	8.20 (6.90–10.75)	9.20 (7.70–11.58)	0.366
ESR peak, mm/h _(23 vs 11)_	40 (22–51)	34 (16–52)	0.793
hsCRP peak, mg/l _(24 vs 12)_	41.70 (13.98–67.80)	55.30 (27.20–95.83)	0.280
PCT peak, ng/ml _(23 vs 12)_	0.03 (0.02–0.05)	0.08 (0.03–0.15)	**0.038**
LDH peak, U/L _(25 vs 12)_	231 (202–316)	312 (232–419)	**0.021**
Fibrinogen peak, g/L _(16 vs 11)_	5.28 ± 1.05	5.47 ± 1.95	0.737
D-dimer peak, μg/mL _(22 vs 12)_	0.62 (0.42–1.37)	1.27 (0.93–2.66)	**0.031**
Albumin min, g/l _(24 vs 12)_	33.9 (30.1–37.9)	29.5 (26.9–30.9)	**0.002**
ALT peak, U/L _(25 vs 12)_	47 (35–69)	53 (37–100)	0.382
AST peak, U/L _(24 vs 12)_	35 (30–46)	44 (40–82)	**0.032**
TB peak, umol/l _(24 vs 12)_	9.9 (8.2–12.4)	13.4 (7.4–15.5)	0.254
BUN peak, mmol/l _(24 vs 12)_	5.2 (3.9–6.1)	5.2 (4.8–7.4)	0.374
Cr peak, umol/l _(25 vs 12)_	80 (62–92)	81 (67–92)	0.570
**Treatment received during hospitalization**			
Oxygen treatment, n (%)	21 (75.0%)	11 (91.7%)	0.396
Cumulative days of oxygen treatment, d _(26 vs 12)_	14 (2–24)	34 (20–45)	** < 0.001**
Maximum inhaled oxygen concentration, % _(23 vs 12)_	33 (21–41)	47 (35–61)	**0.010**
Glucocorticoids, n (%)	14 (50.0%)	10 (83.3%)	0.079
Cumulative days of receiving glucocorticoids, d _(27 vs 12)_	0 (0–9)	12 (6–14)	**0.007**
Total dosage of glucocorticoids, mg‡ _(27 vs 12)_	0 (0–360)	485 (151–838)	**0.024**
Antiviral treatment, n (%)	25 (89.3%)	11 (91.7%)	> 0.999
Antibiotic treatment, n (%)	21 (75.0%)	12 (100%)	0.081
Immunoglobulin therapy, n (%)	9 (32.1%)	5 (41.7%)	0.720
**Clinical data at eight months after discharge**			
BMI, kg/m^2^	24.46 ± 3.59	27.83 ± 4.77	**0.019**
CT score	1 (0–2)	10 (8–16)	** < 0.001**
GGO	10 (35.7%)	11 (91.7%)	**0.002**
Irregular lines	9 (32.1%)	10 (83.3%)	**0.005**
FEV_1_% predicted	103.6 ± 16.1	103.4 ± 18.8	0.981
FVC% predicted	115.1 ± 17.2	113.8 ± 12.8	0.813
FEV_1_/FVC, %	74.5 ± 8.2	74.0 ± 10.6	0.875
TLC% predicted	99.7 ± 8.6	91.4 ± 6.8	**0.005**
RV% predicted	102.4 ± 19.5	84.9 ± 11.9	**0.007**
RV/TLC, %	36.0 ± 8.4	35.1 ± 4.0	0.717
DLCO% predicted	89.9 ± 15.8	80.1 ± 13.2	0.068
DLCO < 80% Predicted, n (%)	6 (21.4%)	7 (58.3%)	**0.032**
DLCO/VA% predicted	92.7 ± 14.4	90.8 ± 15.0	0.713
PCFS scale grade ≥ 1, n (%)	15 (53.6%)	7 (58.3%)	> 0.999
PCFS scale grade ≥ 2, n (%)	7 (25.0%)	2 (16.7%)	0.697
6MWD, m	563 ± 79.3	514 ± 95	0.091

Data were expressed as mean ± SD, median (interquartile range), or No. (%). Comparisons were determined by Student’s t-test, Mann–Whitney U test or Fisher exact tests as appropriate. The actual number of cases was marked behind each index, when there was missing data.

*IL* interleukin, *TNF* tumor necrosis factor, *ESR* erythrocyte sedimentation rate, *hsCRP* high-sensitivity c-reactive protein, *PCT* procalcitonin, *LDH* lactate dehydrogenase, *ALT* alanine aminotransferase, *AST* aspartate aminotransferase, *TB* total bilirubin, *BUN* blood urine nitrogen, *Cr* creatinine, *BMI* body mass index, *GGO* ground glass opacity, *FEV*_*1*_ forced expiratory volume in one second, *FVC* forced vital capacity, *TLC* total lung capacity, *RV* residual volume, *DLCO* diffusion capacity of the lung for carbon monoxide, *DLCO/VA* ratio of carbon monoxide diffusion capacity to alveolar ventilation, *PCFS scale* Post-COVID-19 Functional Status scale, *6MWD* 6-min walk distance.

^£^The CT peak score during hospitalization of one patient with CT score < 5 at follow-up was missing.

^‡^Total dosage of glucocorticoid means accumulative dosage of glucocorticoid converted to prednisone for COVID-19 patients.

#### Patients were divided into two groups according to DLCO at the follow up

As compared with patients with normal DLCO at eight months, patients with abnormal DLCO had longer cumulative days of oxygen treatment (*p* = 0.005), higher rate of receiving glucocorticoids (*p* = 0.040), longer cumulative days of receiving glucocorticoids (*p* = 0.020), and higher total dosage of glucocorticoids used (*p* = 0.031) during hospitalization. At eight months after discharge, patients with abnormal DLCO had higher CT score (*p* = 0.046), and lower MVV% predicted (*p* = 0.019). More details were summarized in Supplementary Table S2.

According to DLCO and DLCO/VA, patients were categorized as group A with DLCO ≥ 80% predicted (n = 27), group B with DLCO < 80% predicted but DLCO/VA ≥ 80% predicted (n = 5), and group C with DLCO and DLCO/VA both < 80% predicted (n = 8). The serum interleukin (IL)-2R, IL-8, and tumor necrosis factor (TNF)-α levels during hospitalization in group C patients were the lowest among the three groups (*p* = 0.023, 0.009, and 0.022, respectively). The TLC% predicted and RV% predicted in group B were lower than those of other two groups (*p* < 0.001, and *p* = 0.010, respectively). Patients in group B had lower X at 5 Hz [− 0.08 ± 0.04 (group A) vs − 0.10 ± 0.02 (group B) vs − 0.05 ± 0.03 (group C), *p* = 0.027], which indicated higher elastic recoil of the peripheral airways respectively. More details were summarized in Table 4 and Supplementary Table S3.

**Table 4:** Characteristics of participants according to DLCO and DLCO/VA at follow-up.

Parameters	DLCO ≥ 80% Predicted (n = 27)	DLCO < 80% Predicted but DLCO/VA ≥ 80% Predicted (n = 5)	DLCO and DLCO/VA both < 80% Predicted (n = 8)	*p* value
Age (years)	54 (39–68)	61 (48–66)	52 (39–70)	0.931
Gender (Female/Male)	12/15	2/3	7/1	0.097
CT peak score £	11 (5–18)	15 (11–23)	14 (10–24)	0.204
Severe cases, n (%)	13 (48.1%)	5 (100%)	7 (87.5%)	**0.023**
**Laboratory data during hospitalization**				
Lymphocyte count min (× 10^9^/L) _(26 vs 5 vs 8)_	0.92 (0.77–1.45)	0.95 (0.74–1.10)	0.54 (0.22–1.28)	0.219
Eosinophil count min (× 10^9^/L) _(25 vs 5 vs 8)_	0.01 (0–0.03)	0.02 (0–0.16)	0 (0–0.02)	0.409
IL-2R peak, U/mL _(17 vs 5 vs 5)_	702 (521–884)	1218 (581–1754) ¶	514 (433–582)	**0.023**
IL-6 peak, pg/mL _(18 vs 5 vs 5)_	14.54 (3.34–43.01)	8.42 (2.84–32.88) ¶	2.43 (1.50–4.22)	0.062
IL-8 peak, pg/mL _(17 vs 5 vs 5)_	15.40 (9.75–37.20)	33.40 (23.25–121.35) ¶	7.20 (5.65–13.25)	**0.009**
TNF-α peak, pg/mL _(17 vs 5 vs 5)_	8.60 (7.70–11.60)	10.10 (8.60–11.90)	5.80 (4.80–7.80)	**0.022**
ESR peak, mm/h _(23 vs 5 vs 6)_	29 (13–51)	45 (19–58)	45 (30–77)	0.406
hsCRP peak, mg/L _(24 vs 5 vs 7)_	38.40 (11.63–77.10)	42.10 (30.75–80.75)	60.60 (27.00–97.30)	0.556
PCT peak, ng/mL _(23 vs 5 vs 7)_	0.04 (0.02–0.06)	0.03 (0.03–0.10)	0.08 (0.04–0.10)	0.125
LDH peak, U/L _(25 vs 5 vs 7)_	241 (202–327)	241 (213–296)	356 (224–428)	0.280
Fibrinogen max, g/L _(18 vs 4 vs 5)_	5.06 ± 1.05	5.71 ± 2.35	6.14 ± 1.89	0.300
D-dimer peak, μg/mL _(22 vs 5 vs 7)_	1.06 (0.48–2.13)	0.83 (0.39–5.26)	1.00 (0.47–1.50)	0.841
Albumin min, g/L _(24 vs 5 vs 7)_	32.6 (29.9–36.7)	30.7 (26.6–35.1)	29.8 (26.9–31.6)	0.186
ALT peak, U/L _(25 vs 5 vs 7)_	51 (39–85)	46 (28–58)	36 (31–120)	0.502
AST peak, U/L _(24 vs 5 vs 7)_	37 (32–64)	31 (25–43)	42 (30–88)	0.308
TB peak, umol/L _(24 vs 5 vs 7)_	10.5 (9.0–15.7)	9.4 (5.7–11.5)	13.2 (9.0–15.6)	0.379
BUN peak, mmol/L _(24 vs 5 vs 7)_	5.2 (4.4–6.3)	5.7 (4.4–5.4)	5.8 (3.0–7.7)	0.594
Cr peak, umol/L _(25 vs 5 vs 7)_	81 (65–92)	82 (60–94)	65 (44–90)	0.532
**Treatment received during hospitalization**				
Oxygen treatment, n (%)	20 (74.1%)	5 (100%)	7 (87.5%)	0.591
Cumulative days of oxygen treatment, d _(25 vs 5 vs 8)_	15 (1–24) †¶	28 (24–39)	35 (11–45)	**0.008**
Maximum inhaled oxygen concentration, % _(22 vs 5 vs 8)_	35 (21–41)	33 (33–47)	49 (35–67)	0.097
Glucocorticoids, n (%)	13 (48.1%)	5 (100%)	6 (75.0%)	0.052
Cumulative days of receiving glucocorticoids, d	0 (0–10) †	11 (10–16)	6 (1–14)	**0.030**
Total dosage of glucocorticoids, mg‡ _(26 vs 5 vs 8)_	0 (0–481)	425 (360–520)	225 (31–938)	0.084
Antiviral treatment, n (%)	24 (88.9%)	4 (80.0%)	8 (100%)	0.552
Antibiotic treatment, n (%)	21 (77.8%)	5 (100%)	7 (87.5%)	> 0.999
Immunoglobulin therapy, n (%)	10 (37.0%)	1 (20.0%)	3 (37.5%)	0.887
**Clinical data at eight months after discharge**				
BMI, kg/m^2^	26.14 ± 4.34	24.29 ± 3.11	23.95 ± 4.30	0.358
Patients with CT score < 5, n (%)	22 (81.5%)	2 (40.0%)	4 (50.0%)	0.067
CT score	2 (0–4)	7 (2–16)	5 (0–13)	0.100
GGO	12 (44.4%)	4 (80.0%)	5 (62.5%)	0.360
Irregular lines	11 (40.7%)	4 (80.0%)	4 (50.0%)	0.249
FEV_1_% predicted	106.8 ± 15.08	95.8 ± 18.3	97.1 ± 19.8	0.194
FVC% predicted	118.0 ± 16.1	104.9 ± 14.1	109.7 ± 13.8	0.142
FEV_1_/FVC, %	74.0 ± 6.0	75.2 ± 3.9	75.1 ± 17.1	0.933
TLC% predicted	99.0 ± 7.4	83.8 ± 6.0§¶	99.6 ± 8.2	** < 0.001**
RV% predicted	98.1 ± 16.1	75.7 ± 10.66§¶	107.5 ± 24.0	**0.010**
RV/TLC, %	35.3 ± 7.2	33.1 ± 3.7	39.0 ± 8.9	0.326
DLCO% predicted	94.9 ± 11.7†¶	70.3 ± 7.9	70.6 ± 7.8	** < 0.001**
DLCO/VA% predicted	98.8 ± 11.4¶	86.7 ± 6.7	73.0 ± 6.8	** < 0.001**
PCFS scale grade ≥ 1	13 (48.1%)	5 (100%)	4 (50.0%)	0.115
PCFS scale grade ≥ 2	6 (22.2%)	2 (40.0%)	1 (12.5%)	0.628
6MWD, m	555 ± 81	535 ± 118	536 ± 82	0.228

Data were expressed as mean ± SD, median (interquartile range), or No. (%). Comparisons were determined by Student’s t-test, Mann–Whitney U test or Fisher exact tests as appropriate. The actual number of cases was marked behind each index, when there was missing data.

^£^The CT peak score during hospitalization of one patient with DLCO and DLCO/VA both < 80% predicted at follow-up was missing.

^‡^Total dosage of systemic glucocorticoid means accumulative dosage of glucocorticoid converted to prednisone for COVID-19 patients**.**

^§^*p* < 0.05 versus DLCO ≥ 80% predicted.

^†^*p* < 0.05 versus DLCO < 80% predicted but DLCO/VA ≥ 80% predicted.

^¶^*p* < 0.05 versus DLCO and DLCO/VA both < 80% predicted.

*IL* interleukin, *TNF* tumor necrosis factor, *ESR* erythrocyte sedimentation rate, *hsCRP* high-sensitivity c-reactive protein, *PCT* procalcitonin, *LDH* lactate dehydrogenase, *ALT* alanine aminotransferase, *AST* aspartate aminotransferase, *TB* total bilirubin, *BUN* blood urine nitrogen, *Cr* creatinine, *BMI* body mass index, *GGO* ground glass opacity, *FEV*_*1*_ forced expiratory volume in one second, *FVC* forced vital capacity, *TLC* total lung capacity, *RV* residual volume, *DLCO* diffusion capacity of the lung for carbon monoxide, *DLCO/VA* ratio of carbon monoxide diffusion capacity to alveolar ventilation, *PCFS scale* Post-COVID-19 Functional Status scale, *6MWD* six-minute walk distance.

### Explore the association between abnormal CT patterns and worse symptoms

To find specific abnormal CT patterns associated to worse symptoms, patients were divided into two groups according to Post-COVID-19 Functional Status (PCFS) scale grade. However, no matter the cut-off value of grouping was set as PCFS scale grade ≥ 1 or 2, there was no significant difference in abnormal CT patterns at eight months after discharge between the two groups (Supplementary Table S4).

### Correlation analyses

Then we analyzed correlations among clinical data during the period having COVID-19, pulmonary function, CT score, and related physiological characteristics at follow up (Supplementary Table S5). CT score after discharge was positively correlated with age (R = 0.417, *p* = 0.008), BMI (R = 0.373, *p* = 0.018), CT peak score (R = 0.769, *p* < 0.001), PCT level (R = 0.367, *p* = 0.030), LDH level (R = 0.371, *p* = 0.024), D-dimer level (R = 0.482, *p* = 0.004), cumulative days of oxygen treatment (R = 0.541, *p* < 0.001), maximum inhaled oxygen concentration (R = 0.623, *p* < 0.001) , cumulative days of receiving glucocorticoids (R = 0.426, *p* = 0.007), and total dosage of glucocorticoids used (R = 0.423, *p* = 0.007) during hospitalization, negatively correlated with lymphocyte count (R = -0.508, *p* = 0.001), and albumin level during hospitalization (R = − 0.515, *p* = 0.001). DLCO% of predicted values was negatively correlated with cumulative days of oxygen treatment (R = − 0.335, *p* = 0.040). DLCO/VA% of predicted values was positively correlated with BMI (R = 0.378, *p* = 0.016), and serum TNF-α level during hospitalization (R = 0.422, *p* = 0.028). The 6MWD was negatively correlated with age (R = − 0.484, *p* = 0.002), BMI (R = − 0.366, *p* = 0.020), PCT level (R = − 0.414, *p* = 0.013), cumulative days of oxygen treatment (R = − 0.377, *p* = 0.020), and maximum inhaled oxygen concentration (R = − 0.377, *p* = 0.026) during hospitalization.

### Logistic regression analyses

Based on univariate analyses, variables with significant differences between patients with normal and abnormal CT (or DLCO) were further analyzed by logistic regression analyses using the forward: LR method. It was found that the increase of CT peak score during hospitalization was the independent risk factor associated with abnormal CT at eight months after discharge (*p* = 0.006, OR 1.370, 95% CI 1.092 to 1.719, Table 5), and longer cumulative days of oxygen treatment was associated with abnormal DLCO at eight months after discharge (*p* = 0.009, OR 1.085, 95% CI 1.021 to 1.154, Table 5).

**Table 5:** Logistic regression analysis of predictors of abnormal CT or DLCO at eight months of follow-up.

	*p* value model	Predictor variables		
**Logistic regression analysis (forward: LR method)**				
Abnormal CT	** < 0.001**	**Variables in the equation**	**OR (95% CI)**	***p* value**
		CT peak score	1.370 (1.092–1.719)	**0.006**
		PCT	9.310 (0.885–97.919)	0.063
		**Variables not in the equation**	**Score**	***p* value**
		Lymphocyte count	0.667	0.414
		LDH	0.118	0.732
		D-dimer	0.008	0.929
		Albumin	3.280	0.070
		AST	0.296	0.586
		Cumulative days of oxygen treatment	2.156	0.142
		Cumulative days of receiving glucocorticoids	2.025	0.155
		BMI	0.179	0.672
Abnormal DLCO	**0.002**	**Variable in the equation**	**OR (95% CI)**	***p* value**
		Cumulative days of oxygen treatment	1.085 (1.021–1.154)	**0.009**
		**Variables not in the equation**	**Score**	***p* value**
		Severity of COVID-19	1.600	0.206
		Cumulative days of receiving glucocorticoids	0.163	0.686

Quantification and assignment: Abnormal CT: “1” for CT score ≥ 5, and “0” for the opposite; PCT: “1” for > 0.05 ng/mL, and “0” for the opposite; Abnormal DLCO: “1” for DLCO% predicted < 80%; “0” for the opposite.

*CI* confidence interval, *PCT* procalcitonin, *LDH* lactate dehydrogenase, *AST* aspartate aminotransferase, *BMI* body mass index.

## Discussion

Since December 2019 a novel coronavirus, now named as SARS-CoV-2, has caused a global COVID-19 pandemic. Although most of the infected persons are asymptomatic or mild patients^8,9^, COVID-19 has led to large number of severe cases due to the huge number of confirmed cases. According to previous reports, anomalies of pulmonary ventilation and diffusion function were noted in a considerable proportion of COVID-19 patients at the time of hospital discharge, especially in patients with severe disease^3^. At 30 days after discharge severe patients still had a higher incidence of DLCO impairment and lower TLC compared with non-severe patients^4^, which indicated that severe patients may need more time to recover and further long time follow-up studies are necessary. At three months after discharge 39 patients (70.9%) still had abnormal CT manifestation and nine patients (16.4%) had impaired DLCO^6^, but only four severe cases were entered in this study. In our study, 25 severe cases were enrolled, a considerable proportion (48%) of severe COVID-19 patients still had abnormalities on DLCO at eight months after discharge. The proportion was higher than that of patients at severity scale 4 (29%) but lower than that of patients at severity scale 5–6 (56%) reported by Cao et al.^7^ at 6 months after discharge. The median CT score of severe cases was 3 (IQR 2–10) at 8 months after discharge which was higher than that of mild cases. Only four severe COVID-19 patients (16%) had completely normal CT, which the proportion was lower than 29.1% reported by Zhao et al.^6^ at three months after discharge. Ten severe COVID-19 patients (40%) still had abnormal CT score above 5. To our knowledge, this is the first study that reported the proportion of patients with abnormal CT on severe COVID-19 cases at over a half of year after discharge. Even at eight months after discharge, severe cases still had a higher incidence of DLCO impairment and lower TLC compared with mild patients. For all patients, patients with abnormal CT had lower TLC% predicted, RV% predicted, higher rate of abnormal DLCO, and a trend of lower 6MWD, indicating that this kind of patients might have potential restrictive ventilation dysfunction, pulmonary diffusion function impairment, and poor exercise tolerance.

Viral pneumonia images are the most common feature of chest CT in patients with COVID-19, mainly present as GGO and consolidative pulmonary opacities^10–12^, as similar as SARS and MERS^13,14^. However, lesions of COVID-19 are more likely to impact bilateral pulmonary and multiple lobs than those of SARS. Previous studies have been reported that SARS have long-term effects on lung function, chest CT scans, and related physiological characteristics in part of survivors, even at one year after discharge^15–18^.

In our study, two of severe cases received N-acetylcysteine (NAC) therapy after discharge from hospital. One of them was treated with NAC for two weeks after discharge, another patient received long-term NAC, and combination inhaled corticosteroids plus long-acting β-agonists (ICS/LABA) therapy due to the comorbidity with COPD. One patient with severe COVID-19 received long-term ICS/LABA therapy due to the comorbidity with asthma, and was planning to receive pirfenidone therapy because of residual abnormal CT manifestation, abnormal DLCO, and abnormal DLCO/VA. Evidence has shown the efficacy of corticosteroid in reducing 28-day mortality in critically ill patients with COVID-19^19^. However, no evidence currently supports or refutes the benefits of corticosteroid or anti-fibrotic agents for patients with persistent symptoms or abnormal CT/DLCO. The efficacy of anti-fibrotic agent, such as pirfenidone, in patients with COVID-19 is mainly speculated based on the pharmacological mechanism and the pathophysiology of COVID-19. Unlike idiopathic pulmonary fibrosis (IPF) or pulmonary fibrosis secondary to autoimmune causes, fibrosis seen in some COVID-19 survivors may not progressive. Therefore, regular follow-up to evaluate the residual pulmonary deficits and the scope of fibrosis is essential to determine the necessity of anti-fibrotic treatment.

Even 12 (48%) severe cases in this study still had abnormal DLCO at over a half of year after discharge, which the proportion was lower than 76.5% reported by Huang et al.^4^ in severe cases at 30 days after discharged, and other lung function indexes, such as FVC and TLC, had the same trends. These results indicated that lung injury caused by COVID-19 in severe cases may have ability to self-rehabilitation, similar to SARS^16^.

However, the rehabilitation process of patients with different severity was variable. Even for two severe cases which had been treated with NIPPV and ICU admission, one of them had basically normal CT and normal DLCO, the other had abnormal CT and DLCO at eight months after discharge (Supplementary Fig. S1). The underlined mechanism is still not well known. As shown in our results, in compared with patients with normal or basically normal CT, patients with abnormal CT had higher CT peak score, higher PCT level, higher LDH level, higher AST level, and longer cumulative days of receiving glucocorticoids and oxygen treatment. Correlation analyses indicated that, CT scores after discharge was positively correlated with age. DLCO% predicted was negatively correlated with cumulative days of oxygen treatment. Logistic regression analyses indicated that the increase of CT peak score was the independent risk factor associated with residual abnormal CT, and longer cumulative days of oxygen treatment was associated with abnormal DLCO at follow-up. These results indicated that COVID-19 had a greater long-term impact on the lung physiology of patients who were older, more severe, and more complicated at the acute phase.

Interestingly, the correlation of BMI with CT abnormalities and pulmonary diffusion function at follow-up was not consistent. Patients with abnormal CT had higher BMI, and BMI positively correlated with CT score, consistent with the finding that obese patients are more likely to develop into severe COVID-19^20^. BMI positively correlated with DLCO/VA% predicted, and had a trend of positive correlation with DLCO% predicted after discharge in this study. In previous studies, the relationship between BMI with pulmonary diffusion function and pulmonary fibrosis has not been sufficiently understood. A study of African Americans with no cardiopulmonary or chest wall disease showed that BMI was negatively correlated with DLCO^21^. Another study on patients with COPD from China showed that BMI was positively correlated with DLCO and DLCO/VA^22^.A series of clinical studies on IPF showed that lower BMI is associated with a poorer outcome of IPF^23–25^, but indexes of pulmonary diffusion function were not observed in these studies. We speculate that better nutritional status may promote the recovery of lung injury from severe COVID-19, as BMI can be considered as an indicator of nutrition.

Surprisingly, six (15%) patients without obvious CT abnormalities also presented an abnormal of DLCO. We think this phenomenon might be caused by microthrombus formation which has been confirmed by the autopsy findings in patients dying of COVID-19^26^. The other option is they may already have abnormal DLCO before COVID-19 disease.

As DLCO could not sufficiently reflect the gas exchange capacity. Then we divided patients into three groups according to DLCO and DLCO/VA. As shown in Table 4, patients with abnormal DLCO but normal DLCO/VA had the lowest TLC% predicted and RV% predicted, markers of restrictive ventilation dysfunction, indicating that for this group of patients the decrease of DLCO was mainly caused by reduced alveolar volume. But pulmonary interstitial or vascular abnormalities caused by COVID-19 might also exist and contribute to the abnormality of DLCO in patients with abnormal DLCO but normal DLCO/VA, because this group of patients had a trend of lower DLCO/VA as compared with patients with normal DLCO (*p* = 0.0502). Surprisingly, we found that patients with abnormal DLCO and DLCO/VA had lowest serum IL-2R, IL-8, and TNF-α levels as compared with the other two groups, although the difference was only significant as compared with patients with abnormal DLCO but normal DLCO/VA. Correlation analyses indicated that DLCO/VA% of predicted values was positively correlated with serum IL-2R and TNF-α level, and had a trend of positive correlation with serum IL-6 level. Previous studies indicated that cytokine storm may contribute to the severity and mortality of COVID-19. In the other hand, previous study had demonstrated that IL-2-deficient mice have an impaired viral clearance capacity in lymphocytic choriomeningitis virus infection^27^. TNF-α has the ability to against virus infection^28^. Animal and vitro experiments indicated that IL-2, IL-2R, IL-8, and IL-6 are involved in the repair process in different tissues after damage^29–32^. All of these indicated that inflammation can be a double-edged sword: inflammation plays an essential role in viral clearance and initiating repair process^33,34^,but hyperinflammation may lead to organs injury and severity of disease. According to the results from this study, we speculate that both hyperinflammation and absent of inflammation could lead to the impairment of pulmonary diffusion function in the recovery stage of COVID-19.

Of note, only 40 patients were recruited. A limited number of cases may lead to biased results, especially in those with strong subjectivity such as SF-36. The limited sample size may also lead us to be unable to find specific abnormal CT patterns associated to worse symptoms and DLCO at follow-up, although our results indicated that abnormal CT was related to abnormal DLCO. We also note that pulmonary function and chest HRCT before SARS-CoV-2 infection are not known, which make it inappropriate to simply attribute abnormal pulmonary function and CT scans to COVID-19. Finally, asymptomatic patients and patients with intubation were not enrolled and only two patients required additional NIPPV treatment were enrolled in this study. The long-term impact of COVID-19 on asymptomatic and critically ill cases could not conclude from this study.

## Conclusions

In conclusion, our study has demonstrated that survivors with severe COVID-19 had higher incidences of DLCO impairment, persistent symptoms in daily life, and higher abnormal CT score as compared with mild cases. The long-term radiological abnormality may relate to old age and the severity of COVID-19. Either absent or excess of inflammation reaction during COVID-19 course would lead to the impairment of pulmonary diffusion function at the recovery stage.

## Methods

### Subjects

In this study, all participants were confirmed by positive SARS-CoV-2 nucleic acid testing results by real-time reverse transcriptase polymerase chain-reaction from January 27, 2020 to March 13, 2020 in Wuhan, China. Severe COVID-19 cases were defined as meeting any of the following^2^: (1) shortness of breath, respiratory rate ≥ 30 times/min; (2) oxygen saturation ≤ 93% at a rest state; (3) partial arterial oxygen pressure/fraction of inspiration O_2_ ≤ 300 mmmHg; (4) the severity of clinical symptoms was aggravating progressively, > 50% lesions progression within 24 to 48 h in lung imaging. Otherwise were defined as mild cases. Exclusion criteria: (1) died before the eight months follow-up visit; (2) unable to follow up because of various serious psychological or physical disorders; (3) missing any of results on chest HRCT, pulmonary function, clinical questionnaires, or 6MWD at the eight months follow-up visit; (4) poor cooperation so that the pulmonary function test results are inaccurate; (5) uncontactable or declined to participate. Finally, our study enrolled 25 survivors with severe COVID-19 and 15 survivors with mild COVID-19.

We collected the clinical data from the onset of COVID-19 and throughout the hospital stay (including epidemiological, demographic, comorbidities characteristics, laboratory data, chest CT scans, and treatment details), and the clinical data at about eight months after discharge (including chest CT scans, characteristics of pulmonary function, and health-related quality of life) of all participants. All the clinical data were checked by two physicians.

All participants were given a written informed consent prior to inclusion. This study was approved by the Ethics Committee of Tongji Hospital, Tongji Medical College, Huazhong University of Science and Technology (IRB ID: TJ-IRB20210115). All methods were carried out in compliance with the Declaration of Helsinki.

### Chest HRCT and image quantification

All participants underwent chest CT scan during the follow-up period. Refer to the method described previously, a radiologic scoring system was used to quantify lung lesions^11^. Briefly, each of the five lung lobes was reviewed for the lesions, such as ground-glass opacification (GGO), interstitial thickening, consolidation, bronchiectasis, irregular interfaces, and so on. According to the percentage area occupied, each lobe was evaluated 0–5 points represent normal performance, lesions involving < 5% of lobe, lesions involving 5–25% of lobe, lesions involving 25–50% of lobe, lesions involving 50–75% of lobe, and lesions involving > 75% of lobe respectively. Then the CT score was reached by summing individual segmental scores.

### Pulmonary function testing

All participants underwent pulmonary function tests in the Pulmonary Function laboratory, Tongji Hospital, Tongji Medical College, Huazhong University of Science and Technology followed by American Thoracic Society/European Respiratory Society guidelines^35^.

The assessment included: forced expiratory volume in one second (FEV_1_), forced vital capacity (FVC), peak expiratory flow (PEF), maximal expiratory flow at 50% of FVC (MEF50), maximal expiratory flow at 25% of FVC (MEF25), maximal midexpiratory flow between 75 and 25% of FVC (MMEF75/25), maximum voluntary ventilation (MVV), TLC, residual volume (RV), DLCO, and ratio of carbon monoxide diffusion capacity to alveolar ventilation (DLCO/VA) measured by means of the single-breath test. The hemoglobin value was also taken for correcting the DLCO.

Impulse oscillation system was used to measure the following parameters: impedance at 5 Hz (Z at 5 Hz), which indicates the total respiratory impedance; resistance in the peripheral airways (Rperipheral); and reactance at 5 Hz corrected by predicted value (X at 5 Hz), which inversely indicates the elastic recoil of the peripheral airways.

### Clinical questionnaires and six-minute walk test (6MWT)

At eight months after discharge all participants answered the Post-COVID-19 Functional Status (PCFS) scale and the MOS 36-item Short-Form Health Survey (SF-36) to assess the impact of severe COVID-19 on health-related quality of life.

PCFS is a simple tool to describe the impact of residual symptoms of COVID-19 on functional status of patients after discharge, which classify patients into 0 to 4 grades. Briefly, grade 0 reflects the absence of any residual symptoms and functional limitation, grade 1 reflects the patient still has persistent physical and (or) psychological symptoms but only has negligible limitations in daily life. From grade 2, residual symptoms and functional limitations are present to an increasing degree. Grade 4 represents the patients suffering from severe functional limitations and requiring assistance from another person due to residual symptoms^36^.

SF-36 consisting of 11 questions and a total of 36 responses that assess physical functioning (PE), role limitation due to physical problems (RP), bodily pain (BP), general health (GH), vitality (VT), social functioning (SF), role limitation due to emotional problems (RE), mental health (MH), and reported health transition (HT). Scores for each item domain range from 0 (worst) to 100 (best). These subscales were then combined to form the physical component summary (PCS) and mental component summary (MCS) scores^37^.

All patients underwent 6MWT without supplemental oxygen, oxygen saturation on pulse oximeter (SpO2) and Borg-scale before and after exercise, and 6MWD were measured as previously described^38^.

### Statistical analyses

Data were expressed as means ± standard deviation (SD) when data were normally distributed or medians with interquartile range (IQR) when data were not normally distributed. Comparisons were determined by Student’s t-test, Mann–Whitney U test or Fisher exact tests as appropriate. Multiple groups were compared using one-way analysis of variance (ANOVA) with a Bonferroni correction (normal data) or a Kruskal–Wallis test with a Dunn intergroup comparison (non-normal data). Spearman’s rank correlation coefficient was used for correlation analyses. We used logistic regression analysis to explore the independent risk factor associated with abnormal CT or DLCO. Statistical significance was considered as *p* < 0.05. SPSS software V.25.0 was used for analyses.

## Supplementary Information

Supplementary Information.

## Data Availability

The datasets generated during and/or analyzed during the current study are available from the corresponding author on reasonable request.

## Abbreviations

COVID-19: Coronavirus disease 2019
SARS-CoV-2: Severe acute respiratory syndrome coronavirus 2
CFR: Case-fatality rate
SARS: Severe acute respiratory syndrome
MERS: Middle East respiratory syndrome
DLCO: Diffusion capacity of the lung for carbon monoxide
TLC: Total lung capacity
6MWD: Six-minute walk distance
HRCT: High-resolution computed tomography
GGO: Ground-glass opacification
FEV_1_: Forced expiratory volume in one second
FVC: Forced vital capacity
PEF: Peak expiratory flow
MEF50: Maximal expiratory flow at 50% of FVC
MEF25: Maximal expiratory flow at 25% of FVC
MMEF75/25: Maximal midexpiratory flow between 75 and 25% of FVC
MVV: Maximum voluntary ventilation
RV: Residual volume
DLCO/VA: Ratio of carbon monoxide diffusion capacity to alveolar ventilation
Z at 5 Hz: Impedance at 5 Hz
Rperipheral: Resistance in the peripheral airways
X at 5 Hz: Reactance at 5 Hz corrected by predicted value
6MWT: Six-minute walk test
PCFS: The Post-COVID-19 Functional Status
SF-36: The MOS 36-item Short-Form Health Survey
SD: Standard deviation
IQR: Interquartile range
ANOVA: One-way analysis of variance
BMI: Body mass index
COPD: Chronic obstructive pulmonary disease
NIPPV: Noninvasive positive pressure ventilation
LDH: Lactate dehydrogenase
PCT: Procalcitonin
IL: Interleukin
TNF: Tumor necrosis factor
NAC: N-acetylcysteine
ICS/LABA: Combination inhaled corticosteroids plus long-acting β-agonists
IPF: Idiopathic pulmonary fibrosis
