# Supplementary material for: Eight months follow-up study on pulmonary function, lung radiographic, and related physiological characteristics in COVID-19 survivors
Source: Sci Rep. 2021 Jul 5;11:13854. doi: 10.1038/s41598-021-93191-y (PMC8257634; doi:10.1038/s41598-021-93191-y)
Supplement: Supplementary file 1 — Supplementary Information. [file 41598_2021_93191_MOESM1_ESM.docx]

**Eight months follow-up study on pulmonary function, lung radiographic, and related physiological characteristics in COVID-19 survivors**

Shengding Zhang^1,2^ (MD), Wenxue Bai^1,2^ (MD), Junqing Yue^1,2^ (MD), Lu Qin^1,2^ (MD), Cong Zhang^1,2^ (MD), Shuyun Xu^1,2^ (MD), Xiansheng Liu^1,2^ (MD), Wang Ni^1,2🖂^ (BS), Min Xie^1,2🖂^ (MD)

^1^Department of Respiratory and Critical Care Medicine, Tongji Hospital, Tongji Medical College, Huazhong University of Science and Technology, Wuhan, China.

^2^Key Laboratory of Respiratory Diseases, National Ministry of Health of the People's Republic of China and National Clinical Research Center for Respiratory Disease, Wuhan, China.

**Correspondence to:** Prof. Min Xie, Department of Respiratory and Critical Care Medicine, Tongji Hospital, Tongji Medical College, Huazhong University of Science and Technology, Wuhan 430030, China; e-mail: xie_m@126.com; Wang Ni, Department of Respiratory and Critical Care Medicine, Tongji Hospital, Tongji Medical College, Huazhong University of Science and Technology, Wuhan 430030, China; e-mail: niwang@tjh.tjmu.edu.cn.

**
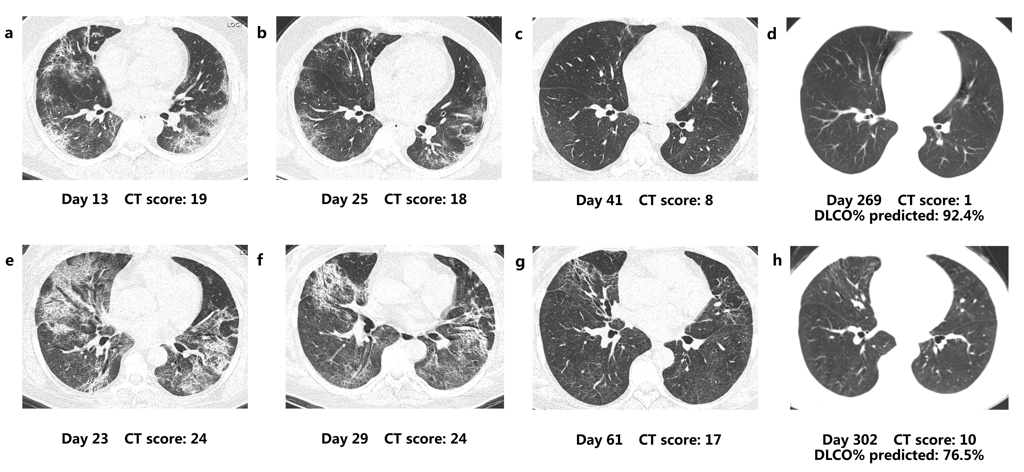
**

**Figure S1. Serial HRCTs of two COVID-19 survivors who received pressure ventilation.**

(a-d) Serial HRCTs of a 37-year-old man with confirmed COVID-19: (a) First HRCT in hospital on February 12, 2020 (13 days after symptoms onset). CT image shows diffuse bilateral GGOs, consolidative pulmonary opacities, and irregular lines. (b) Part of lesions were absorbed over 12 days. (c) On March 11, 2020, lesions were further absorbed. (d) On November 21, 2020, residual fibrotic streaks were observed.

(e-h) Serial HRCTs of a 47-year-old woman with confirmed COVID-19: (e) First HRCT in hospital on February 16, 2020 (23 days after symptoms onset). CT image shows diffuse bilateral GGOs, consolidative pulmonary opacities, and irregular lines. (f) Part of lesions were further consolidated, part of lesions were absorbed. (g) On March 25, 2020, HRCT showed that previous lesions were further absorbed. GGOs and fibrotic streaks were observed. (h) On November 21, 2020, residual fibrotic streaks and GGOs were observed.

**Table S1. Characteristics of participants according to CT scanning at follow-up**

| **Parameters** | | **CT score at eight months after discharge** | | ***p* value** |
| --- | --- | --- | --- | --- |
|  |  | **< 5（n = 28）** | **≥ 5 (n = 12)** |  |
| Age (years) | | 51 (38-68) | 65 (55-65) | 0.256 |
| Gender (Female/Male) | | 15/13 | 6/6 | > 0.999 |
| CT peak score £ | | 9 (5-14) | 23 (14-24) | **< 0.001** |
| Severe cases, n (%) | | 15 (53.6%) | 10 (83.3%) | 0.152 |
| **Laboratory data during hospitalization** | |  |  |  |
| Lymphocyte count min (× 10^9^/L) _(27 vs 12)_ | 1.08 (0.73-1.50) | 0.77 (0.46-0.93) | **0.003** |  |
| Eosinophil count min (× 10^9^/L) _(26 vs 12)_ | 0.01 (0-0.03) | 0 (0-0.02) | 0.309 |  |
| IL-2R peak, U/ml _(17 vs 10)_ | 536 (440-755) | 792 (498-1109) | 0.183 |  |
| IL-6 peak, pg/ml _(18 vs 10)_ | 4.20 (2.40-25.79) | 8.89 (3.20-39.15) | 0.493 |  |
| IL-8 peak, pg/ml _(17 vs 10)_ | 15.40 (7.90-43.70) | 16.85 (11.80-30.38) | 0.776 |  |
| TNF-α peak, pg/ml _(17 vs 10)_ | 8.20 (6.90-10.75) | 9.20 (7.70-11.58) | 0.366 |  |
| ESR peak, mm/h _(23 vs 11)_ | 40 (22-51) | 34 (16-52) | 0.793 |  |
| hsCRP peak, mg/l _(24 vs 12)_ | 41.70 (13.98-67.80) | 55.30 (27.20-95.83) | 0.280 |  |
| PCT peak, ng/ml _(23 vs 12)_ | 0.03 (0.02-0.05) | 0.08 (0.03-0.15) | **0.038** |  |
| LDH peak, U/L _(25 vs 12)_ | 231 (202-316) | 312 (232-419) | **0.021** |  |
| Fibrinogen peak, g/L _(16 vs 11)_ | 5.28 ± 1.05 | 5.47 ± 1.95 | 0.737 |  |
| D-dimer peak, μg/mL _(22 vs 12)_ | 0.62 (0.42-1.37) | 1.27 (0.93-2.66) | **0.031** |  |
| Albumin min, g/l _(24 vs 12)_ | 33.9 (30.1-37.9) | 29.5 (26.9-30.9) | **0.002** |  |
| ALT peak, U/L _(25 vs 12)_ | 47 (35-69) | 53 (37-100) | 0.382 |  |
| AST peak, U/L _(24 vs 12)_ | 35 (30-46) | 44 (40-82) | **0.032** |  |
| TB peak, umol/l _(24 vs 12)_ | 9.9 (8.2-12.4) | 13.4 (7.4-15.5) | 0.254 |  |
| BUN peak, mmol/l _(24 vs 12)_ | 5.2 (3.9-6.1) | 5.2 (4.8-7.4) | 0.374 |  |
| Cr peak, umol/l _(25 vs 12)_ | 80 (62-92) | 81 (67-92) | 0.570 |  |
| **Treatment received during hospitalization** | |  |  |  |
| Oxygen treatment, n (%) | 21 (75.0%) | 11 (91.7%) | 0.396 |  |
| Cumulative days of oxygen treatment, d _(26 vs 12)_ | 14 (2-24) | 34 (20-45) | **< 0.001** |  |
| Maximum inhaled oxygen concentration, % _(23 vs 12)_ | 33 (21-41) | 47 (35-61) | **0.010** |  |
| Glucocorticoids, n (%) | 14 (50.0%) | 10 (83.3%) | 0.079 |  |
| Cumulative days of receiving glucocorticoids, d _(27 vs 12)_ | 0 (0-9) | 12 (6-14) | **0.007** |  |
| Total dosage of glucocorticoids, mg‡ _(27 vs 12)_ | 0 (0-360) | 485 (151-838) | **0.024** |  |
| Antiviral treatment, n (%) | 25 (89.3%) | 11 (91.7%) | > 0.999 |  |
| Antibiotic treatment, n (%) | 21 (75.0%) | 12 (100%) | 0.081 |  |
| Immunoglobulin therapy, n (%) | 9 (32.1%) | 5 (41.7%) | 0.720 |  |
| **Clinical data at eight months after discharge** | | |  | |
| BMI, kg/m^2^ | 24.46 ± 3.59 | 27.83 ± 4.77 | **0.019** |  |
| CT score | 1 (0-2) | 10 (8-16) | **< 0.001** |  |
| **CT abnormalities, n (%)** | |  |  |  |
| GGO | 10 (35.7%) | 11 (91.7%) | **0.002** |  |
| Irregular lines | 9 (32.1%) | 10 (83.3%) | **0.005** |  |
| Consolidation | 0 (0%) | 0 (0%) | > 0.999 |  |
| Interlobular septal thickening | 0 (0%) | 0 (0%) | > 0.999 |  |
| Subpleural line | 1 (3.6%) | 1 (7.7%) | 0.515 |  |
| Reticular pattern | 0 (0%) | 2 (16.7%) | 0.085 |  |
| **Pulmonary function** | |  |  |  |
| FEV_1_% predicted | 103.6 ± 16.1 | 103.4 ± 18.8 | 0.981 |  |
| FVC% predicted | 115.1 ± 17.2 | 113.8 ± 12.8 | 0.813 |  |
| FEV_1_/FVC, % | 74.5 ± 8.2 | 74.0 ± 10.6 | 0.875 |  |
| PEF% predicted | 108.4 ± 18.5 | 115.4 ± 16.3 | 0.259 |  |
| MEF50% predicted | 71.8 ± 20.1 | 74.5 ± 30.1 | 0.740 |  |
| MEF25% predicted | 51.7 ± 24.8 | 48.0 ± 21.6 | 0.658 |  |
| MMEF75/25% predicted | 60.1 ± 23.2 | 63.8 ± 25.3 | 0.840 |  |
| MVV% predicted | 113.4 ± 16.3 | 109.4 ± 20.5 | 0.523 |  |
| Z at 5Hz% predicted | 97.5 ± 20.5 | 102.0 ± 26.3 | 0.559 |  |
| Rperipheral, kPa/(L/s) | 0.22 ± 0.09 | 0.25 ± 0.12 | 0.460 |  |
| X at 5Hz corrected by predicted value, kPa/(L/s) | -0.07 ± 0.04 | -0.08 ± 0.05 | 0.761 |  |
| TLC% predicted | 99.7 ± 8.6 | 91.4 ± 6.8 | **0.005** |  |
| RV% predicted | 102.4 ± 19.5 | 84.9 ± 11.9 | **0.007** |  |
| RV/TLC, % | 36.0 ± 8.4 | 35.1 ± 4.0 | 0.717 |  |
| DLCO% predicted | 89.9 ± 15.8 | 80.1 ± 13.2 | 0.068 |  |
| DLCO < 80% Predicted, n (%) | 6 (21.4%) | 7 (58.3%) | **0.032** |  |
| DLCO/VA% predicted | 92.7 ± 14.4 | 90.8 ± 15.0 | 0.713 |  |
| **The Post-COVID-19 functional status scale** | |  |  |  |
| PCFS scale grade ≥ 1, n (%) | 15 (53.6%) | 7 (58.3%) | > 0.999 |  |
| PCFS scale grade ≥ 2, n (%) | 7 (25.0%) | 2 (16.7%) | 0.697 |  |
| **SF-36** | |  |  |  |
| PF | 93 (80-100) | 90 (82-95) | 0.588 |  |
| RP | 100 (100-100) | 100 (19-100) | 0.513 |  |
| BP | 100 (74-100) | 94 (74-100) | 0.895 |  |
| GH | 70 (48-97) | 74 (65-95) | 0.493 |  |
| VT | 80 (59-90) | 90 (73-95) | 0.145 |  |
| SF | 75 (63-100) | 100 (63-100) | 0.200 |  |
| RE | 100 (100-100) | 100 (0-100) | 0.252 |  |
| MH | 80 (69-88) | 88 (62-91) | 0.613 |  |
| HT | 50 (25-50) | 50 (32-50) | 0.320 |  |
| PCS | 85 (68-96) | 89 (61-96) | 0.936 |  |
| MCS | 84 (67-93) | 90 (53-97) | 0.488 |  |
| 6MWD, m | | 563 ± 79.3 | 514 ± 95 | 0.091 |

Data were expressed as mean ± SD, median (interquartile range), or No. (%). Comparisons were determined by Student’s t-test, Mann-Whitney U test or Fisher exact tests as appropriate. The actual number of cases was marked behind each index, when there was missing data.

£ The CT peak score during hospitalization of one patient with CT score < 5 at follow-up was missing.

‡ Total dosage of systemic glucocorticoid means accumulative dosage of glucocorticoid converted to prednisone for COVID-19 patients**.**

**Abbreviations:** IL, interleukin; TNF, tumor necrosis factor; ESR, erythrocyte sedimentation rate; hsCRP, high-sensitivity c-reactive protein; PCT, procalcitonin; LDH, lactate dehydrogenase; ALT, alanine aminotransferase; AST, aspartate aminotransferase; TB, total bilirubin; BUN, blood urine nitrogen; Cr, creatinine; BMI, body mass index; GGO, ground glass opacity; FEV_1_, forced expiratory volume in one second; FVC, forced vital capacity; PEF, peak expiratory flow; MEF50, maximal expiratory flow at 50% of FVC; MEF25, maximal expiratory flow at 25% of FVC; MMEF75/25, maximal midexpiratory flow between 75% and 25% of FVC; MVV, maximum voluntary ventilation; Z at 5Hz, impedance at 5 Hz, which indicates the total respiratory impedance; Rperipheral, resistance in the peripheral airways; X at 5Hz, reactance at 5 Hz corrected by predicted value, which inversely indicates the elastic recoil of the peripheral airways; TLC, total lung capacity; RV, residual volume; DLCO, diffusion capacity of the lung for carbon monoxide; DLCO/VA, ratio of carbon monoxide diffusion capacity to alveolar ventilation; PCFS scale, Post-COVID-19 Functional Status scale; PF, physical functioning; RP, role limitation due to physical problems; BP, bodily pain; GH, general health; VT, vitality; SF, social functioning; RE, role limitation due to emotional problems; MH, mental health; HT, reported health transition; PCS, physical component summary; MCS, mental component summary; 6MWD, six-minute walk distance.

**Table S2. Pulmonary function, chest CT, and related physiological characteristics according to DLCO at follow-up**

| **Parameters** | | **DLCO ≥ 80% Predicted (n = 27)** | **DLCO < 80% Predicted (n = 13)** | ***p* value** |
| --- | --- | --- | --- | --- |
| **CT abnormalities, n (%)** | |  |  |  |
| GGO | 12 (44.4%) | 9 (69.2%) | 0.186 |  |
| Irregular lines | 11 (40.7%) | 8 (61.5%) | 0.314 |  |
| Consolidation | 0 (0%) | 0 (0%) | > 0.999 |  |
| Interlobular septal thickening | 0 (0%) | 0 (0%) | > 0.999 |  |
| Subpleural line | 2 (7.4%) | 0 (0%) | > 0.999 |  |
| Reticular pattern | 1 (3.7%) | 1 (7.7%) | > 0.999 |  |
| **Pulmonary function** | |  |  |  |
| FEV_1_% predicted | 106.8 ± 15.1 | 96.6 ± 18.5 | 0.069 |  |
| FVC% predicted | 118.0 ± 16.1 | 107.9 ± 13.5 | 0.056 |  |
| FEV_1_/FVC, % | 74.0 ± 6.0 | 75.1 ± 13.2 | 0.707 |  |
| PEF% predicted | 113.7 ± 18.5 | 103.9 ± 15.3 | 0.104 |  |
| MEF50% predicted | 73.8 ± 20.3 | 70.0 ± 29.0 | 0.625 |  |
| MEF25% predicted | 46.9 ± 17.0 | 58.2 ± 33.2 | 0.162 |  |
| MMEF75/25% predicted | 62.8 ± 18.7 | 52.1 ± 32.3 | 0.928 |  |
| MVV% predicted | 116.6 ± 16.7 | 103.0± 15.9 | **0.019** |  |
| Z at 5Hz% predicted | 100.6 ± 23.0 | 95.1 ± 20.6 | 0.469 |  |
| Rperipheral, kPa/(L/s) | 0.23 ± 0.11 | 0.24 ± 0.09 | 0.788 |  |
| X at 5Hz corrected by predicted value, kPa/(L/s) | -0.08 ± 0.04 | -0.07 ± 0.04 | 0.429 |  |
| TLC% predicted | 99.0 ± 7.4 | 93.5 ± 10.7 | 0.064 |  |
| RV% predicted | 98.1 ± 16.1 | 95.3 ± 25.1 | 0.671 |  |
| RV/TLC, % | 35.3 ± 7.2 | 36.7 ± 7.7 | 0.569 |  |
| DLCO% predicted | 94.9 ± 11.7 | 70.5 ± 7.5 | **< 0.001** |  |
| DLCO/VA% predicted | 98.8 ± 11.4 | 78.3 ± 9.4 | **< 0.001** |  |
| **The Post-COVID-19 functional status scale, n (%)** | |  | |  |
| PCFS scale grade ≥ 1 | 13 (48.1%) | 9 (69.2%) | 0.312 |  |
| PCFS scale grade ≥ 2 | 6 (22.2%) | 3 (23.1%) | > 0.999 |  |
| **SF-36** | |  |  |  |
| PF | 90 (80-100) | 90 (78-98) | 0.660 |  |
| RP | 100 (100-100) | 100 (0-100) | 0.219 |  |
| BP | 100 (74-100) | 74 (68-100) | 0.388 |  |
| GH | 72 (57-97) | 67 (55-92) | 0.845 |  |
| VT | 85 (70-90) | 80 (63-93) | 0.725 |  |
| SF | 75 (63-100) | 63 (63-100) | 0.854 |  |
| RE | 100 (100-100) | 100 (0-100) | 0.400 |  |
| MH | 84 (72-88) | 80 (60-90) | 0.513 |  |
| HT | 50 (25-50) | 50 (25-50) | 0.674 |  |
| PCS | 88 (69-97) | 82 (60-95) | 0.435 |  |
| MCS | 87 (79-94) | 81 (52-95) | 0.554 |  |
| 6MWD, m | | 555 ± 81 | 535 ± 92 | 0.499 |

Data were expressed as mean ± SD, median (interquartile range), or No. (%). Comparisons were determined by Student’s t-test, Mann-Whitney U test or Fisher exact tests as appropriate.

**Abbreviations:** GGO, ground glass opacity; FEV_1_, forced expiratory volume in one second; FVC, forced vital capacity; PEF, peak expiratory flow; MEF50, maximal expiratory flow at 50% of FVC; MEF25, maximal expiratory flow at 25% of FVC; MMEF75/25, maximal midexpiratory flow between 75% and 25% of FVC; MVV, maximum voluntary ventilation; Z at 5Hz, impedance at 5 Hz, which indicates the total respiratory impedance; Rperipheral, resistance in the peripheral airways; X at 5Hz, reactance at 5 Hz corrected by predicted value, which inversely indicates the elastic recoil of the peripheral airways; TLC, total lung capacity; RV, residual volume; DLCO, diffusion capacity of the lung for carbon monoxide; DLCO/VA, ratio of carbon monoxide diffusion capacity to alveolar ventilation; PCFS scale, Post-COVID-19 Functional Status scale; PF, physical functioning; RP, role limitation due to physical problems; BP, bodily pain; GH, general health; VT, vitality; SF, social functioning; RE, role limitation due to emotional problems; MH, mental health; HT, reported health transition; PCS, physical component summary; MCS, mental component summary; 6MWD, six-minute walk distance.

**Table S3. Pulmonary function, chest CT, and related physiological characteristics according to DLCO and DLCO/VA at follow-up**

| **Parameters** | | **DLCO ≥ 80% Predicted (n = 27)** | **DLCO < 80% Predicted but DLCO/VA ≥ 80% Predicted (n = 5)** | **DLCO and DLCO/VA both < 80% Predicted (n = 8)** | ***p* value** |
| --- | --- | --- | --- | --- | --- |
| **CT abnormalities, n (%)** | |  |  |  |  |
| GGO | 12 (44.4%) | 4 (80.0%) | 5 (62.5%) | 0.360 |  |
| Irregular lines | 11 (40.7%) | 4 (80.0%) | 4 (50.0%) | 0.249 |  |
| Consolidation | 0 (0%) | 0 (0%) | 0 (0%) | > 0.999 |  |
| Interlobular septal thickening | 0 (0%) | 0 (0%) | 0 (0%) | > 0.999 |  |
| Subpleural line | 2 (7.4%) | 0 (0%) | 0 (0%) | > 0.999 |  |
| Reticular pattern | 1 (3.7%) | 0 (0%) | 1 (12.5%) | 0.550 |  |
| **Pulmonary function** | |  |  |  |  |
| FEV_1_% predicted | 106.8 ± 15.08 | 95.8 ± 18.3 | 97.1 ± 19.8 | 0.194 |  |
| FVC% predicted | 118.0 ± 16.1 | 104.9 ± 14.1 | 109.7 ± 13.8 | 0.142 |  |
| FEV_1_/FVC, % | 74.0 ± 6.0 | 75.2 ± 3.9 | 75.1 ± 17.1 | 0.933 |  |
| PEF% predicted | 113.7 ± 18.5 | 107.6 ± 16.8 | 101.5 ± 14.9 | 0.227 |  |
| MEF50% predicted | 73.8 ± 20.3 | 66.8 ± 22.4 | 71.9 ± 33.9 | 0.829 |  |
| MEF25% predicted | 46.9 ± 17.0 | 47.4 ± 24.4 | 65.0 ± 37.6 | 0.160 |  |
| MMEF75/25% predicted | 62.8 ± 18.7 | 60.1 ± 26.2 | 63.4 ± 37.3 | 0.969 |  |
| MVV% predicted | 116.6 ± 16.7 | 101.2 ± 20.3 | 104.2 ± 13.9 | 0.064 |  |
| Z at 5Hz% predicted | 100.6 ± 23.0 | 109.7 ± 23.2 | 86.0 ± 13.1 | 0.131 |  |
| Rperipheral, kPa/(L/s) | 0.23 ± 0.11 | 0.27 ± 0.12 | 0.21 ± 0.07 | 0.565 |  |
| X at 5Hz corrected by predicted value, kPa/(L/s) | -0.08 ± 0.04 | -0.10 ± 0.02¶ | -0.05 ± 0.03 | **0.027** |  |
| TLC% predicted | 99.0 ± 7.4 | 83.8 ± 6.0§¶ | 99.6 ± 8.2 | **< 0.001** |  |
| RV% predicted | 98.1 ± 16.1 | 75.7 ± 10.66§¶ | 107.5 ± 24.0 | **0.010** |  |
| RV/TLC, % | 35.3 ± 7.2 | 33.1 ± 3.7 | 39.0 ± 8.9 | 0.326 |  |
| DLCO% predicted | 94.9 ± 11.7†¶ | 70.3 ± 7.9 | 70.6 ± 7.8 | **< 0.001** |  |
| DLCO/VA% predicted | 98.8 ± 11.4¶ | 86.7 ± 6.7 | 73.0 ± 6.8 | **< 0.001** |  |
| **The Post-COVID-19 functional status scale, n (%)** | | | |  |  |
| PCFS scale grade ≥ 1 | 13 (48.1%) | 5 (100%) | 4 (50.0%) | 0.115 |  |
| PCFS scale grade ≥ 2 | 6 (22.2%) | 2 (40.0%) | 1 (12.5%) | 0.628 |  |
| **SF-36** | |  |  |  |  |
| PF | 90 (80-100) | 95 (78-100) | 88 (78-95) | 0.721 |  |
| RP | 100 (100-100) | 100 (88-100) | 50 (0-100) | 0.142 |  |
| BP | 100 (74-100) | 74 (68-100) | 87 (65-100) | 0.662 |  |
| GH | 72 (57-97) | 65 (50-92) | 77 (53-95) | 0.926 |  |
| VT | 85 (70-90) | 80 (75-93) | 78 (55-94) | 0.833 |  |
| SF | 75 (63-100) | 63 (51-100) | 82 (63-100) | 0.895 |  |
| RE | 100 (100-100) | 100 (50-100) | 100 (0-100) | 0.510 |  |
| MH | 84 (72-88) | 80 (62-90) | 84 (49-91) | 0.779 |  |
| HT | 50 (25-50) | 25 (25-50) | 50 (31-50) | 0.302 |  |
| PCS | 88 (69-97) | 90 (71-95) | 75 (50-96) | 0.535 |  |
| MCS | 87 (79-94) | 81 (61-95) | 75 (49-96) | 0.816 |  |
| 6MWD, m | | 555 ± 81 | 535 ± 118 | 536 ± 82 | 0.228 |

Data were expressed as mean ± SD, median (interquartile range), or No. (%). Comparisons were determined by Student’s t-test, Mann-Whitney U test or Fisher exact tests as appropriate.

§, *p* < 0.05 versus DLCO ≥ 80% predicted.

†, *p* < 0.05 versus DLCO < 80% predicted but DLCO/VA ≥ 80% predicted.

¶, *p* < 0.05 versus DLCO and DLCO/VA both < 80% predicted.

**Abbreviations:** GGO, ground glass opacity; FEV_1_, forced expiratory volume in one second; FVC, forced vital capacity; PEF, peak expiratory flow; MEF50, maximal expiratory flow at 50% of FVC; MEF25, maximal expiratory flow at 25% of FVC; MMEF75/25, maximal midexpiratory flow between 75% and 25% of FVC; MVV, maximum voluntary ventilation; Z at 5Hz, impedance at 5 Hz, which indicates the total respiratory impedance; Rperipheral, resistance in the peripheral airways; X at 5Hz, reactance at 5 Hz corrected by predicted value, which inversely indicates the elastic recoil of the peripheral airways; TLC, total lung capacity; RV, residual volume; DLCO, diffusion capacity of the lung for carbon monoxide; DLCO/VA, ratio of carbon monoxide diffusion capacity to alveolar ventilation; PCFS scale, Post-COVID-19 Functional Status scale; PF, physical functioning; RP, role limitation due to physical problems; BP, bodily pain; GH, general health; VT, vitality; SF, social functioning; RE, role limitation due to emotional problems; MH, mental health; HT, reported health transition; PCS, physical component summary; MCS, mental component summary; 6MWD, six-minute walk distance.

**Table S4. Abnormal CT patterns according to PCFS scale grade at follow-up**

| **CT abnormalities** | **PCFS scale grade < 1（n = 18）** | **PCFS scale grade ≥ 1（n = 22）** | ***p* value** |
| --- | --- | --- | --- |
| GGO | 9 (50.0%) | 12 (54.5%) | > 0.999 |
| Irregular lines | 7 (38.9%) | 12 (54.5%) | 0.360 |
| Consolidation | 0 (0%) | 0 (0%) | > 0.999 |
| Interlobular septal thickening | 0 (0%) | 0 (0%) | > 0.999 |
| Subpleural line | 0 (0%) | 2 (9.1%) | 0.492 |
| Reticular pattern | 1 (5.6%) | 1 (4.5%) | > 0.999 |
| **CT abnormalities** | **PCFS scale grade < 2（n = 31）** | **PCFS scale grade ≥ 2（n = 9）** | ***p* value** |
| GGO | 16 (51.6%) | 5 (55.6%) | > 0.999 |
| Irregular lines | 13 (41.9%) | 6 (66.7%) | 0.265 |
| Consolidation | 0 (0%) | 0 (0%) | > 0.999 |
| Interlobular septal thickening | 0 (0%) | 0 (0%) | > 0.999 |
| Subpleural line | 1 (3.2%) | 1 (11.1%) | 0.404 |
| Reticular pattern | 2 (6.5%) | 0 (0%) | > 0.999 |

Data were expressed as No. (%). Comparisons were determined by Fisher exact tests.

**Abbreviations:** PCFS scale, Post-COVID-19 Functional Status scale; GGO, ground glass opacity.

**Table S5. Correlations between clinical data during hospitalization and CT score, DLCO, and 6MWD at follow-up**

|  | **CT score** | **DLCO% predicted** | **DLCO/VA% predicted** | **6MWD** |
| --- | --- | --- | --- | --- |
| **Age (n = 40)** | R = 0.417  *p* = 0.008 | R = -0.020  *p* = 0.903 | R = 0.063  *p* = 0.699 | R = -0.484  *p* = 0.002 |
| **BMI (n = 40)** | R = 0.373  *p* = 0.018 | R = 0.291  *p* = 0.069 | R = 0.378  *p* = 0.016 | R = -0.366  *p* = 0.020 |
| **CT peak score (n = 39)** | R = 0.769  *p* < 0.001 | R = -0.178  *p* = 0.278 | R = 0.013  *p* = 0.939 | R = -0.231  *p* = 0.157 |
| **Lymphocyte count (n = 39)** | R = -0.508  *p* = 0.001 | R = 0.256  *p* = 0.116 | R = 0.103  *p* = 0.533 | R = 0.115  *p* = 0.487 |
| **IL-2R (n = 27)** | R = 0.271  *p* = 0.172 | R = 0.008  *p* = 0.967 | R = 0.236  *p* = 0.235 | R = 0.069  *p* = 0.732 |
| **IL-6 (n = 28)** | R = -0.017  *p* = 0.932 | R = 0.292  *p* = 0.132 | R = 0.314  *p* = 0.104 | R = 0.038  *p* = 0.850 |
| **TNF-α (n = 27)** | R = 0.246  *p* = 0.215 | R = 0.244  *p* = 0.220 | R = 0.422  *p* = 0.028 | R = 0.152  *p* = 0.450 |
| **PCT (n = 35)** | R = 0.367  *p* = 0.030 | R = -0.250  *p* = 0.147 | R = -0.253  *p* = 0.142 | R = -0.414  *p* = 0.013 |
| **LDH (n = 37)** | R = 0.371  *p* = 0.024 | R = -0.302  *p* = 0.070 | R = -0.238  *p* = 0.156 | R = -0.050  *p* = 0.768 |
| **D-dimer (n = 34)** | R = 0.482  *p* = 0.004 | R = 0.058  *p* = 0.745 | R = 0.144  *p* = 0.415 | R = -0.074  *p* = 0.676 |
| **Albumin (n = 36)** | R = -0.515  *p* = 0.001 | R = 0.259  *p* = 0.127 | R = 0.021  *p* = 0.901 | R = 0.233  *p* = 0.171 |
| **Cumulative days of oxygen treatment (n = 38)** | R = 0.541  *p* < 0.001 | R = -0.335  *p* = 0.040 | R = -0.208  *p* = 0.210 | R = -0.377  *p* = 0.020 |
| **Maximum inhaled oxygen concentration (n = 35)** | R = 0.623  *p* <0.001 | R = -0.257  *p* = 0.137 | R = -0.215  *p* = 0.215 | R = -0.377  *p* = 0.026 |
| **Cumulative days of receiving glucocorticoids (n = 39)** | R = 0.426  *p* = 0.007 | R = -0.261  *p* = 0.109 | R = -0.080  *p* = 0.628 | R = -0.059  *p* = 0.723 |
| **Total dosage of glucocorticoids used (n = 39)** | R = 0.423  *p* = 0.007 | R = -0.292  *p* = 0.071 | R = -0.112  *p* = 0.496 | R = -0.030  *p* = 0.854 |

Spearman R-values and *p*-values were indicated. The actual number of cases was marked behind each index.

**Abbreviations:** BMI, body mass index; IL, interleukin; TNF, tumor necrosis factor; PCT, procalcitonin; LDH, lactate dehydrogenase; DLCO, diffusion capacity of the lung for carbon monoxide; DLCO/VA, ratio of carbon monoxide diffusion capacity to alveolar ventilation; 6MWD, six-minute walk distance.
